# Antioxidant and Anti-Inflammatory Properties of Mushroom-Based Food Additives and Food Fortified with Them—Current Status and Future Perspectives

**DOI:** 10.3390/antiox14050519

**Published:** 2025-04-26

**Authors:** Agata Michalska, Małgorzata Sierocka, Beata Drzewiecka, Michał Świeca

**Affiliations:** 1 Department of Biochemistry and Food Chemistry, University of Life Sciences, Skromna Str. 8, 20-704 Lublin, Poland; malgorzata.sierocka@up.lublin.pl; 2 Sub-Department of Pathophysiology, Department of Preclinical Veterinary Sciences, Faculty of Veterinary Medicine, University of Life Sciences, 20-033 Lublin, Poland; beata.drzewiecka@up.lublin.pl

**Keywords:** antioxidants, bioaccessibility, mushrooms, functional food, food fortification, microencapsulation

## Abstract

Due to an aging population and the prevalence of illnesses associated with modern lifestyles, mushrooms, well known for their nutritional value and health-promoting properties, are becoming an increasingly important part of the diet. They are consumed in various forms, including food, nutraceuticals, and dietary supplements. A relatively new trend involves incorporating mushrooms or their components as additives and supplements to enhance the quality and functionality of traditional food products. The processing and preservation methods of fresh mushrooms can significantly impact the activity of resulting powders, extracts, or other functional forms used in food additives, supplements, and fortified foods. The functional benefits of mushrooms are frequently attributed to their antioxidant and anti-inflammatory properties. However, to date, the literature lacks comprehensive reviews that consolidate existing knowledge on mushroom-based food additives and products enriched with them. Therefore, this review aims to compile and methodically analyze the existing data in this field, identify existing knowledge gaps, and outline future perspectives for the development and application of such products. Special attention is given to food supplementation with microencapsulated additives, which represent a promising form of functional powders. All these aspects are evaluated in terms of their antioxidant and anti-inflammatory properties. Finally, future perspectives on improving the health benefits of food through mushroom-based additives are discussed.

## 1. Introduction

In recent years, leading a healthy lifestyle has become a significant trend in society. As a result, diet has become an essential means of providing bioactive compounds that contribute to the proper functioning of the human body or enhance its performance [1]. Consumer preferences have thus shifted toward functional products rich in active substances, including antioxidants and compounds with anti-inflammatory properties. Numerous studies indicate that disruptions in the body’s homeostasis contribute to the development of various lifestyle diseases, such as obesity, diabetes, cardiovascular diseases, neurodegenerative disorders (e.g., Alzheimer’s and Parkinson’s), and cancer [2,3,4,5,6,7]. In response to that, researchers are exploring new natural alternative sources of bioactive substances, with mushrooms occupying a significant place [8,9,10].

Nowadays, mushrooms have become versatile and sustainable ingredients in the food industry. They are recognized for enhancing organoleptic properties and as valuable ingredients with high nutritional value and significant health-promoting and functional benefits. Due to their low-fat content, mushrooms are classified as low-calorie foods, rich in high-quality protein and dietary fiber [11,12]. The content of protein varies depending on the mushroom species and for example, its content in jelly leaf mushrooms (*Tremella mesenterica* Retz.) or yellow blusher mushrooms (*Tricholoma sejunctum* (Sowerby) Quél.) ranges from 8.5 g to 36.5 g per 100 g of dry matter, while popular culinary species such as porcini mushrooms (*Boletus edulis* Bull.) and white button mushroom (*Agaricus bisporus* (J.E. Lange) Imbach) contain about 25% dry matter [13]. As mentioned, mushrooms are also a valuable source of dietary fiber (including chitin, hemicelluloses, mannans, and β-glucans), whose content in commonly consumed, commercially available mushroom species ranges from 27% in king trumpet mushrooms (*Pleurotus eryngii* (DC.) Quél) to 32% in shiitake mushrooms (*Lentinula edodes* (Berk.) Pegler)) [14]. In addition to their high nutrient content, mushrooms also contain a diverse profile of active substances with a wide range of bioactivities, as confirmed by in vitro and in vivo studies [15,16,17,18]. These include vitamins (B-group vitamins and vitamin D) [19], macro- and microelements (selenium, zinc, copper, potassium, calcium, and iron) [20,21,22], polysaccharides (e.g., lentinan from shiitake mushrooms (*Lentinula edodes*), schizophyllan from splitgill mushroom (*Schizophyllum commune* Fr.) [20] and terpenoids (ganoderic acid from Reishi mushroom (*Ganoderma lucidum* (Curtis) P. Karst.), and lentinellic acid from *Lentinellus micheneri* ((Berk. & M.A. Curtis) Pegler)) [22]. Additionally, mushrooms contain lectins and sterols (ergosterol from *Agaricus bisporus* or brassicasterol from *Pleurotus eryngii*) [23]. Some studies also report the presence of phenolic compounds in mushroom fruiting bodies, such as p-hydroxybenzoic acid from Maitake mushroom (*Grifola frondosa* (Dicks.) Gray) and p-coumaric acid from white button mushroom (*Agaricus bisporus* (J.E. Lange) Imbach) [21]. These substances exhibit a range of health-promoting effects, including hypoglycemic activity (inotodiol from Chaga mushroom (*Inonotus obliquus* (Ach. ex Pers.) Pilát)) [24], hypocholesterolemic and immunomodulatory effects (pleuromutilin from *Clitopilus passeckerianus* (Pilát) Singer, applanoxidic acid from beeswax bracket (*Ganoderma pfeifferi* Bres.)) [25,26], as well as anticancer properties (methylantcinate B from cinnamon antrodia mushroom (*Antrodia cinnamomea*), xylomannan from saffron milk cap *(Lactarius deliciosus* (L.) Pers.)) [27,28].

**Figure 1:**
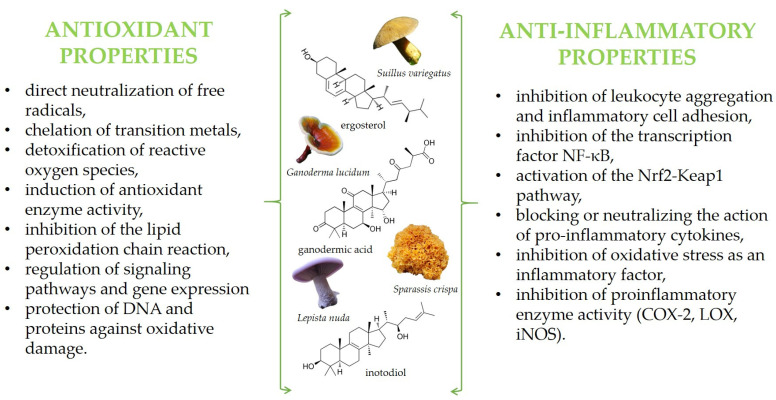
The main active compounds in mushrooms exhibiting antioxidant and anti-inflammatory properties, along with the proposed mechanisms of action.

Among these activities are also antioxidant and anti-inflammatory properties, which, so far, have been summarized in some reviews [11,29,30,31,32]. Briefly, mushroom bioactive components exhibit protective effects at various stages of the oxidation and inflammation processes, employing diverse mechanisms of action. Depending on their chemical structure, they can function in both hydrophilic and hydrophobic environments. These compounds may act directly as free radical scavengers—neutralizing free radicals by donating electrons or hydrogen atoms—or indirectly by inhibiting their production, for example, through the suppression of pro-oxidative or pro-inflammatory enzyme activity. Additionally, they can enhance enzymatic antioxidant defenses by stimulating the activity of enzymes such as superoxide dismutase (SOD), catalase (CAT), and glutathione peroxidase (GPx). Finally, mushroom bioactives may function as cell-signaling molecules or gene expression modulators, leading to alterations in gene regulation. Some proposed mechanisms of action are illustrated in the graphic below (Figure 1).

In contrast to isolated ingredients, the activity of mushroom extracts or fractions used in functional additives production is also created or modified by the interactions with formulation co-ingredients and, once incorporated into a product, by interactions with components of the food matrix [33,34]. Both components (e.g., fat, dietary fiber) and environment (e.g., pH, digestive enzymes) can significantly influence their bioavailability, stability, and overall antioxidant and anti-inflammatory activity. For example, lipids may enhance the solubility and absorption of lipophilic antioxidants (e.g., ergosterol derivatives) [35], while dietary fiber may bind and trap antioxidants, slowing their release and absorption. On the other hand, the same dietary fiber can act synergistically with mushroom β-glucans in terms of antioxidant and immunomodulatory properties, as well as the promotion of microbiota growth [36,37].

The unique properties of mushrooms have been actively explored in recent years, and the existing knowledge has been systematically compiled in numerous review papers [14,38,39,40], which, however, largely focuses on evaluating isolated bioactive ingredients or multi-component fractions, mainly in in vitro models. However, the effectiveness of mushroom components has also been confirmed through in vivo studies, although primarily in animal models [41]. So far, there is currently a lack of translational studies and clearly defined dose–response relationships in in vivo human models, especially in humans, which is largely due to legal regulations limiting the introduction of mushroom-derived substances into the human diet. However, in some countries, such as Japan, mushroom components are recognized as valuable therapeutic agents (e.g., Krestin containing Polysaccharide K). The efficacy of PSK, for instance, has been evaluated in clinical trials involving patients with gastric, colorectal, or lung cancers [42,43,44]. Nevertheless, further clinical studies are necessary to assess both the effectiveness of medicinal mushrooms within the complex human body and the safety of their use.

Previous reviews on mushroom applications in food technology and human nutrition have mainly addressed isolated compounds or fractions from the fruiting bodies or mycelium of edible and medicinal species. These reviews primarily focus on their potential bioactivities, including health-promoting properties and medical applications, as well as their chemical composition, nutritional value, and role in the development of functional foods. Unfortunately, the current literature lacks scientific papers describing the anti-inflammatory and antioxidant properties of mushroom-based food additives and products enriched with them. Therefore, the objective of this review is to systematically compile and analyze the available data in this field, identify existing knowledge gaps, and propose future directions for the development and application of these products.

## 2. Materials and Methods

In this study, a mixed methodology was carried out, including a bibliometric analysis of papers obtained from the PubMed, ScienceDirect, Scopus, and Google Scholar databases (10 January 2025). The entered query string included the terms “mushrooms”, “antioxidant activity”, “anti-inflammatory activity”, “food additives”, “food fortification”, and “encapsulation”. To summarize the most important findings, original studies, systematic reviews, and meta-analyses that investigated the antioxidant and anti-inflammatory properties of mushroom-based food additives and food fortified with them were included (eligibility criteria). Studies not directly related to the topic, as well as opinion articles, editorials, reports, and conference abstracts, were excluded (Supplementary Figure S1, Prisma diagram [45]). The results of the selected studies were critically summarized and analyzed, highlighting key findings, limitations, and implications for future research.

## 3. Mushrooms in the Food Industry—Mushroom-Based Foods and Their Role in Reducing Oxidative Stress and Inflammation

Due to their seasonal availability and susceptibility to spoilage, mushrooms are rarely consumed in their raw form. For this reason, various processes are applied to extend their shelf life while ensuring adequate consumer quality. However, this processing affects the quantitative and qualitative composition of the product, which is reflected in changes to its bioactive properties.

### 3.1. Mushroom Fruit Body and Its Processed Forms

Before consumption, mushrooms are often subjected to thermal processing methods such as cooking, freezing, or frying. These processes can lead to the oxidation of active compounds and may cause tissue damage. Both of these factors also significantly influence the composition and activity of antioxidants and inflammation modulators (Table 1). Sun et al. [46] tested the effect of five different cooking treatments, including steaming (8 min, under atmospheric pressure), pressure cooking (5 min, high pressure), microwaving (1.5 min, 900 W), frying (3 min, 160 °C), and boiling (10 min, 100 °C) on the antiradical (^•^OH (hydroxyl) and DPPH (2,2-difenylo-1-pikrylohydrazyl)) and reducing properties of porcini mushrooms (Boletus). They found that boiling significantly decreased the phenolic content (particularly *p*-hydroxybenzoic and gallic acids), leading to a subsequent three-fold reduction in the ability to quench ^•^OH radicals. It is important to note that the observed changes were both species-dependent (varying among the studied Boletus) and method-dependent (depending on the type of antioxidant assay). For example, the ability to scavenge ^•^OH radicals in boiled samples of *B. aureus* and *B. edulis* was three times lower compared to the raw samples, whereas, in *B. pinophilus* and *B. badius*, this activity remained nearly unchanged. In contrast, the effect of thermal treatment on DPPH scavenging activity was minimal, regardless of mushroom species. However, in the case of reducing power, significant decreases were generally observed. Adamska and Felisiak [47] observed a decrease in the ability to scavenge DPPH free radicals and in reducing properties in blanched (10 min, tap water, 100 °C) and further processed (frying-20 min, oil; stewing—40 min, tap water; boiling—40 min, tap water) chicken mushrooms (*Laetiporus sulphureus* (Bull.) Murrill), likely due to a reduced content of total polyphenols (by 20%), including flavonoids (by 42%). On the other hand, except for blanching and cooking, the culinary treatments had no significant effect on, or actually improved, the ABTS (2,2′-azino-bis (3-ethylbenzothiazoline-6-sulfonic acid radical) and DPPH scavenging properties of shiitake mushrooms. Blanching (1 min boiling water) caused an approximate 40% decrease in ABTS and DPPH scavenging activity, while cooking resulted in an undesirable reduction of up to 70% [48]. In turn, Ramos et al. [49] examined the impact of various cooking methods, such as grilling (6 min 100 °C), deep frying (3 min 160 °C), boiling (10 min 100 °C), and microwaving (1.5 min 1000 W), on the antioxidant capacity of white button mushrooms. Their study revealed that cooking and frying significantly reduced polyphenol content (by 48% and 38%, respectively) and antioxidant activity in the ABTS test (by 52% and 41%, respectively), as well as in the ferric reducing antioxidant power test (FRAP) (by 47% and 23%, respectively). In contrast, all cooking methods enhanced the ability to scavenge free radicals in the DPPH test, with grilling and microwave cooking showing more than a twofold increase in activity.

Mushrooms are also traditionally subjected to drying processes, which not only preserve them but also significantly alter their composition and health benefits. For example, Villalobos-Pesos et al. [50] demonstrated that different drying methods (hot air drying, freeze drying, and microwave-vacuum drying) can notably reduce the polyphenolic content and antioxidant properties of oyster mushrooms (*Pleurotus ostreatus* (Jacq.) P. Kumm.). Among the dried samples, the highest free radical scavenging activity (measured via the DPPH test) was observed in mushrooms dried with hot air (HAD) at 40 °C and 80 °C (around 90% recovery). In contrast, freeze-dried samples showed the lowest activity (approximately 42%). Similarly, the air-drying of scaber stalk (*Leccinum scabrum* (Bull.) Gray) and lion’s mane (*Hericium erinaceus* (Bull.) Pers.) resulted in a reduction in radical scavenging ability (as measured using ABTS and DPPH tests) in the following order: fresh samples > 40 °C (28 h) > 70 °C (7 h) [51].

**Table 1:** Influence of processing or preservation methods on antioxidant and/or anti-inflammatory properties and examples.

Mushroom and Processing	Impact on Properties Compared to Unprocessed	Ref.
*Boletus* sp. Boiling (2 h, 90 °C)	Hydroxyl radical scavenging (^•^OH) of methanolic extract ↓, DPPH scavenging activity of methanolic extract ↓	[46]
*Enoki*, *Shitake* Microwave boiling (9000 W, 10 min)	NO production in induced RAW 264.7 macrophages ↓, TNFα production in induced RAW 264.7 macrophages ↓	[52]
*Pleurotus citrinopileatus* Drying (20–22 °C, 72 h)	EGT Content Determination ↑, hydroxyl radical scavenging ability ↑, peroxy free radicals (ROO^•^), hydroxyl free radicals (^•^OH), and peroxynitrites (ONOO^•^) ↑	[53]
*Lentinus edodes* Canning (85 °C medium and heating in 121 °C, 30 min)	Scavenging of hydrogen peroxide ↓, scavenging of ABTS radical anion ↓, Assessment of the antioxidant action by the deoxyribose assay ↓	[54]
*Lepista nuda* Freezing in a fluid bed tunnel (−30 °C, 4 min)	Inhibition of peroxidation in the lipid system ↓, improvement of the oxidative stability of olive oil ↑,	[54]
*Pleurotus eryngii* Grilling (100 °C, 6 min)	Total polyphenols content ↑, ABTS scavenging ↑, DPPH radical scavenging ability ↑, the ferric-reducing ability FRAP ↑	[49]
*Lentinula edodes* Frying (160 °C, 3 min)	DPPH radical scavenging ability ↓, total polyphenols content ↓, the ferric-reducing ability FRAP ↓, ABTS scavenging ↓	[49]

NO—nitric oxide; TNFα—tumor necrosis factor α; EGT—ergothioneine; ↑—an increase in properties/activity; ↓—a decrease in properties/activity.

Lactic acid fermentation is a widely used and cost-effective method for preserving mushrooms, as it enhances their flavor profile, preserves them, and usually positively affects their composition. As previously mentioned, the fermentation process also influences the content of bioactive compounds and antioxidant activity. Jabłońska-Ryś et al. [55] investigated the impact of different strains of lactic acid bacteria (LAB) as starter cultures on the polyphenol content and antioxidant potential of oyster mushrooms and common chanterelles (*Cantharellus cibarius* Fr.). They demonstrated that fermented mushrooms had a lower total polyphenol content (1.23 mg gallic acid equivalents (GAE)/g d.w. for oyster mushrooms and 1.08 mg GAE/g d.w. for common chanterelles) compared to fresh fruiting bodies (2.10 mg GAE/g d.w. and 1.88 mg GAE/g d.w., respectively). In both species, a decrease in reducing potential (measured using the FRAP test) and free radical scavenging ability (measured using the DPPH test) was observed. In the common chanterelle, for both methods, an approximate 44% reduction in activities was found, while oyster mushrooms experienced about a 59% and 44% decrease in DPPH and FRAP tests, respectively. The study also indicated that the choice of starter culture influenced the antioxidant properties of the mushrooms, particularly in chanterelles. Fermentation with *Lactiplantibacillus plantarum* led to lower antiradical activity (20.87 µmol Trolox equivalents (TE)/g d.w.) compared to samples fermented with other *Lactobacillus* strains.

Processing and preservation methods have a significant impact on the bioactive composition of mushrooms as well as their antioxidant and anti-inflammatory properties. The nature of these changes is not uniform and depends both on the mushroom species and the type of treatment applied. In the case of thermal processing, such as boiling, frying, or microwaving, there is often a substantial loss of active compounds and a weakening of antioxidant capacity. Generally, boiling leads to the most significant degradation of these properties. It is worth noting, however, that some methods, e.g., grilling or short microwave exposure, can even increase antiradical activity. Drying processes, while effective in prolonging shelf life, also usually negatively affect antioxidant capacity. It has been proven that processing may cause the degradation, conversion, or release of active forms of valuable bioactive compounds, which is reflected in the diversification of antioxidant and anti-inflammatory properties. Although such technological approaches are necessary to ensure the stability and safety of the mushrooms, they come with unavoidable quality compromises.

### 3.2. Supplements and Nutraceuticals

Dietary supplements and nutraceuticals have gained popularity in recent years due to their ability to enhance and support various bodily functions, as well as their ease of consumption and appealing product forms. They exhibit a range of beneficial properties, including immunomodulatory, anti-obesity, and anti-diabetic effects, and usually contain bioactive compounds derived from mushrooms, such as polysaccharides, triterpenes, lipids, and peptides. These components may serve as the main active ingredients, contribute to the product matrix, or influence functionality (e.g., controlled release). Due to their specific nature, these food products are commonly available in the form of powders (dried and crushed fruiting bodies or isolated fractions (e.g., Organic Mega 10 Mushroom Powder, MicroIngredients^®^); Shiitake Mushroom Extract Powder, BulkSupplements.com^®^), capsules and tablets (e.g., Triple Mushroom Combo, LancoNutrition^®^), or tinctures and liquid extracts (e.g., Mushroom Wisdom, Maitake D-Fraction, Pro 4X, iHerb^®^). While many of these products are standardized, with manufacturers clearly specifying the levels of active compounds, others are not, lacking disclosed composition details and providing only general claims about potential health benefits.

In the study by Sharpe et al. [56], the antioxidant properties of commercial tinctures from six edible mushrooms (chaga, maitake, reishi, shiitake, lion’s mane, and turkey tail) provided by Catskill Fungi^®^ were compared with those of laboratory-prepared counterparts. The studied extracts exhibited different antioxidant capacities depending on species and extract origin (in the Oxygen Radical Absorbance Capacity test (ORAC), e.g., 0.05 to 1.8 μmol TE/mg (laboratory extracts) vs. 0.023 to 0.63 μmol TE/mg (commercial tinctures)). Using five different antioxidant assays (ORAC, Nanoceria Reducing Antioxidant Capacity (NanoCerac), DPPH radical scavenging, Total Phenolic Content (TPC), and FRAP), chaga displayed the highest activity, followed by maitake. The study demonstrated detectable antioxidant activity in all commercially available hydro-alcoholic extracts, even after an average of 9 months of storage at room temperature. At the same time, it was shown that the antioxidant activity of the mushrooms undergoes drastic changes by the time of purchase (ca. 50% decrease after 4 months).

Antioxidant capacity of commercial food supplements (powdered mushrooms) from shaggy mane (*Coprinus comatus* (O.F. Müll.) Pers.), caterpillar fungus (*Ophiocordyceps sinensis* (Berk.)), and reishi purchased in Serbian pharmacies were tested after Soxhlet extraction (menthol) [57]. All tested extracts exhibited the ability to quench DPPH radicals with IC50 (half maximal inhibitory concentration) values of 0.17, 0.4.8, and 0.24 mg/mL for caterpillar fungus, reishi, and shaggy mane, respectively (IC50 for α-tocopherol was 0.002 mg/mL). *O. sinensis* and *C. comatus* effectively scavenged ^•^OH radicals (IC50 values 0.38 and 0.31 mg/mL, respectively), while reishi does not have this activity. All supplements had comparable reducing properties (A700 ranged from 0.53 to 0.65 for 5 mg/mL extract). The study comparing two food supplements containing *Cordyceps militaris* (FS1, Mountain Rose Herbs, Eugene, OR, USA; powdered mycelia; FS2, Noomadic Herbals, Markham, ON, Canada; extract standardized for 30% D-glucans content) showed that they have a comparable ability to quench DPPH radicals, although significantly lower than that observed in fruiting bodies from commercial cultivation [58]. Also, the aqueous suspension of commercial *Coprinus comatus* powder (100%, product for human use; lekovito.com) administered to animals with oxidative stress induced with carbon tetrachloride and alloxan showed antioxidative potential (improved activity of enzymatic defense), evidenced by an increase in antioxidative status of liver reflected in the prevention of histological changes in liver cross-sections [59].

Despite the declaration of specific properties, their levels may significantly differ in dietary supplements available on the market. This fact has been confirmed by Zrnić Ćirić et al. [60], who revealed considerable differences in β-glucan content (mushroom component responsible for pro-health properties) and antiradical properties among the 13 studied food supplements commercially available in Serbian pharmacies and health food stores. For example, supplements based on Chaga ((extract 4:1) 300 mg; vitamin C 100 mg; recommended daily dose: 2–3 times per day) had a 200-times greater antioxidant properties (FRAP test) than Reishi/Shiitake preparation (extract powder (10:1) 45 mg; Reishi powder 150 mg; Shiitake extract powder (4:1) 75 mg; recommended daily dose: 4 times per day). These variations stem from the mushroom species, the presence of other active ingredients, the formulation types, and the recommended dosing regimens.

Mushroom-based dietary supplements and nutraceuticals are gaining increasing popularity among consumers; however, an analysis of the available data reveals significant variation in composition, activity, and quality among commercial preparations. The antioxidant and anti-inflammatory properties are often highlighted on product labels; however, this information is rarely confirmed by reliable, targeted studies on the preparations themselves. Instead, it is typically based on the previously determined activity of individual ingredients. Moreover, even standardized preparations may lose their properties over time. From the perspective of consumer safety and food quality, the current situation in the market highlights an urgent need for standardization and regulation.

### 3.3. Mushroom-Based Food Additives and Food Supplemented with Them

Mushroom-based food additives are used to preserve or enhance organoleptic characteristics, modify health-promoting properties and nutritional value, and ensure desirable textural qualities. They are available in various forms (e.g., powders, extracts), which significantly influence their functionality (Table 2). Below, we review the most commonly used groups of these additives, highlighting their role in enhancing the antioxidant and anti-inflammatory capacity of food. Fortification with dried, powdered fruit bodies is the most common method of enhancing food with bioactive compounds. Various drying techniques are used in powder production, and the impact of these technologies on the content, quality, and activity of bioactive compounds has been previously reviewed [61]. Below, we present successful examples of improving the antioxidant and anti-inflammatory properties of food through fortification with powdered fruit bodies. In a study by Ibrahim et al. [62], oyster mushrooms were sliced, dipped in a 0.5% citric acid solution, and then dehydrated at 45 °C for 12 h in a thermostatically controlled hot air oven before being processed into powder. The resulting powder, intended for use in bakery products, contained 6.02 mg GAE/g of phenolics and 1.7 mg of vitamin D per 100 g, with reducing properties (FRAP test) of 0.42 mg GAE/g. The fortification of waffles and breadsticks with this additive (0–3%) led to a 2.7- and 2.8-fold increase in antiradical properties (DPPH test), respectively. A similar effect was also observed for the reducing properties (FRAP test), with a 2.6- and 3.3-fold increase, respectively. Additionally, dried powders of Maitake (*Grifola frondosa*) and Enoki (*Flammulina filiformis*) mushrooms were successfully incorporated into tagliatelle pasta at concentrations of 0–10% (*w*/*w*). These powders proved to be valuable sources of radical scavengers, metal chelators, and inhibitors of pro-inflammatory enzyme activity. The addition of these mushroom powders resulted in a significant increase in the ability to scavenge ABTS radicals, showing a dose-independent effect. A dose-dependent effect of fortification was observed in terms of reducing power, and, importantly, the compounds demonstrated high in vitro bioaccessibility. Pasta enriched with 10% powdered Enoki and Maitake mushrooms showed an increased ability to inhibit lipoxygenase (LOX) activity (by 53% and 55%, respectively) and cyclooxygenase-2 (COX-2) activity (by 14% and 18%, respectively). Overall, LOX inhibitors were effectively released from the food matrix during simulated gastrointestinal digestion, with the bioaccessible fractions exhibiting significantly higher activity compared to those extracted using phosphate-buffered saline (PBS). The absence of COX-2 inhibitory activity in PBS extracts suggests that the responsible compounds may be bound to the food matrix. These findings highlight the digestive tract’s role as an effective extraction system and underscore the importance of including such testing in food quality assessments [63]. The addition of powdered white button (*Agaricus bisporus*), shiitake (*Lentinula edodes*), and porcini (*Boletus edulis*) mushrooms (sliced and dried at 55 °C for 24 h) increased the antioxidant potential of durum wheat pasta. The powdered additives from white button mushroom and porcini mushroom exhibited comparable activities, with values of 7.5 µmol/g d.w. and 62 µmol TE/g d.w. in the DPPH and ORAC tests, respectively. In contrast, shiitake mushroom (SM) was less active by approximately 20% and 35% in these tests. Fortifying the pasta by replacing semolina with mushroom powder (0–15% *w*/*w*) resulted in a dose-independent increase in activity in the DPPH test, although the increase was lower than expected based on the individual supplement results. At a 5% substitution level, no significant change in activity was observed, regardless of the type of mushroom used. However, with shiitake mushrooms, changes were noted only in the 15% substitution samples. In the ORAC test, the activity increased across the entire range of fortification. The pasta with white button mushroom showed the highest increase, although this was disproportionate to the amount of supplement used, with results outperforming expectations based on the additive’s activity [64].

An emerging trend in food additive production involves the use of liquid extracts or their dried counterparts. This strategy enables the effective isolation of bioactive compounds and simplifies the standardization process. The resulting additives are characterized by significantly higher concentrations of active ingredients, allowing for smaller doses to be used. This approach also ensures the rapid and uniform dispersion of functional components within the food matrix and improves both bioaccessibility and bioavailability. Moreover, extracts can be further processed to obtain specific fractions with unique compositions and bioactivities. In some cases, liquid additives may help avoid technological and sensory modifications of food products, which are often unavoidable when mushrooms are added in their fresh or dried forms. On the other hand, liquid forms may have a shorter shelf life (a high-water content may affect microbiological stability), and they are more difficult to store at an industrial scale. While studies on the properties of liquid mushroom extracts are frequently conducted [65,66,67,68], reports on their application and observed effects in food are still relatively rare.

The incorporation of lyophilized aqueous acetone extracts (0–5%) of Boletus mushrooms characterized by the high content of tocopherols, ascorbic acid, and phenolic acids significantly improved the antioxidant properties of beef patties [69]. The fortification of burgers with 5% of extract increased radical scavenging properties (DPPH test), reducing power, and lowered lipid peroxidation (Thiobarbituric Acid Reactive Substances (TBARSs)) by 76%, 49%, and 83%, respectively. Moreover, the positive effects were also kept after 8-day storage—a change of the mentioned activities to 54%, 4%, and 38%, respectively. The fortification of Omega-3-supplemented milk with a hydroalcoholic extract from almond mushroom (*Agaricus subrufescens* Peck) residues caused a significant decrease in lipid oxidation. This strategy enabled the production of functional milk containing highly bioaccessible radical scavengers (ABTS test) and compounds with reducing properties (FRAP test). After in vitro digestion, these activities in the enriched milk increased by 55% and 165%, respectively, compared to raw milk. The authors suggested that the polyphenols introduced into milk are bioavailable and can act against the free radicals present in the human body, providing antioxidant activity [70]. Two types of extracts from Shiitake by-products (water and 50% ethanolic) were incorporated into the fermented sausage recipe. This resulted in significantly higher antioxidant activity (FRAP, chelating power, DPPH test) and an increased inhibitory capacity against lipid oxidation (TBARS test). Moreover, the effects of the ethanolic extract were more efficient and remained more stable during storage (40 days) [71]. Crude water-soluble polysaccharides (cWSP) from fruiting bodies of oyster mushroom were added (0–0.5%) to the yogurts. Fortifying led to a slight increase in the antioxidant capacity in the FRAP assay (up to ~12%) and ABTS assay (up to ~23%) [72].

**Table 2:** The effect of food supplementation/fortification with different forms of mushroom-based additives on their antioxidant and/or anti-inflammatory properties—examples.

Industry and Product	Mushroom and Its Form	Antioxidant or Anti-Inflammatory Effect	Ref.
Dairy, yogurt	Dried ethanolic *A. blazei* ergosterol-rich extract (1.4%)	increases in the antiradical (DPPH test) and reducing capacity (FRAP test), positive effect accelerated during cold storage	[73]
Dairy, spreadable cheese	β-Glucan (0.4%) *Pleurotus* *ostreatus*	no effect on the antiradical capacity (DPPH test) and metal chelating ability during 21-day storage, slight increase in the superoxide scavenging activity (up to 10% in the 21st day of storage)	[74]
Bakery, white bread	dried shiitake, porcini, white button mushroom (0–15%)	enhanced the DPPH radical scavenging properties (up to 12-fold), increased oxygen radical absorbance (ORAC) capacity (up to 8-fold)	[75]
Bakery, white bread	freeze-dried *Pleurotus eryngii* and *Cantharellus cibarius* (0–15%)	enhanced the DPPH radical scavenging properties (up to 7-fold in 15% *C. cibarius* bread), dose-independent increase the ability to quench ABTS radicals	[76]
Meat industry, fish burgers	steamed *Pleurotus ostreatus,* (0–20%)	dose-independent, slight improvement in reducing properties (FRAP test), dose-dependent increase in the ability to quench ABTS radicals (up to 30% with 20% substitution)	[77]
Meat industry, sausages	ergothioneine-enriched *Flammulina velutipes* extract	reduction in lipid peroxidation during storage (by 35% after 2 weeks), Protection of protein carbonylation (by 30% after 2 weeks)	[78]
Beverages, isotonic drink	β-glucan rich extract from *Tremella fuciformis* (0–10%)	increased radical scavenging properties (DPPH test), Lack of anti-inflammatory effects (nitric oxide and TNF-α cytokine production test in Lipopolysaccharide (LPS)-induced RAW 264.7 macrophages)	[79]
Beverages, kombucha	fermented *Ganoderma* *lucidum* (Reishi)	significantly lower radical scavenging properties (compared to tea and coffee), High ability in the β-Carotene bleaching test	[80]
Pasta, noodle	wood ear mushroom (*Auricularia cornea*) powder (0–5%)	increased radical scavenging properties (DPPH test) by 44%, increases reducing capacity (FRAP test) by 48%	[81]

Current research confirms the significant potential of mushroom-based food additives, with the creation of antioxidant and anti-inflammatory properties of products. While many studies report enhanced antioxidant activity following the fortification, these effects are often dependent on the mushroom species, the form of the additive (powder vs. extract), the applied dose, and the type of food product. Moreover, some studies suggest a lack of proportionality between the amount of the additive and the observed effect. This may be due to the limited bioavailability of active compounds or their interactions with the food matrix. Although the functional potential of mushroom-based additives is undeniable, their actual industrial implementation remains limited. This may be attributed to inconsistent sensory outcomes, difficulties in ensuring reproducibility between raw material batches, the low stability of certain additive forms, and the lack of regulatory standards and guidelines. Further research is also needed on the bioavailability and bioaccessibility of active compounds, as well as the effects of long-term consumption.

### 3.4. Microencapsulated Mushroom-Based Additives

#### 3.4.1. Microencapsulation Process

Microencapsulation is an advanced technology that has recently gained significant attention in the food industry, particularly for the development of functional foods. This technique involves encapsulating nutritional and bioactive compounds within a protective material to safeguard them from degradation during food processing, including exposure to high temperatures, oxygen, or light. Additionally, microencapsulation enhances the stability and bioavailability of bioactive compounds while facilitating their controlled release to the target site. This process also plays a crucial role in masking undesirable flavors and aromas, thereby improving the sensory attributes of the final food product [82,83,84].

Microencapsulation produces microspheres ranging in size from 1 μm to 100 μm, where the encapsulated bioactive compound is referred to as the core, and the surrounding protective shell is known as the encapsulant. The selection of an appropriate encapsulating material depends on its rheological properties, ability to stabilize and disperse the active compound, and overall structural integrity. Additionally, the encapsulant should provide sufficient mechanical strength, remain chemically inert with the core material, and facilitate controlled release at a specific location and time. Common encapsulating materials include carbohydrates (e.g., starch, modified starch, maltodextrin, cellulose, and cyclodextrin), proteins (e.g., gluten and gelatin), and gums (e.g., agar, gum acacia, and carrageenan) [82,85]. Microencapsulation can be achieved through various techniques, which are broadly categorized into physical (e.g., spray drying, lyophilization), physicochemical (e.g., coacervation, liposome formation), and chemical methods (e.g., polymerization and molecular inclusion complexation) [86,87].

Given the growing interest in mushrooms’ health benefits, microencapsulation facilitates or even directly enables the incorporation of different bioactive fractions/compounds into diverse food formulations, enhancing their application in the functional food industry [88,89,90].

#### 3.4.2. Microencapsulated Food Additives

**Figure 2:**
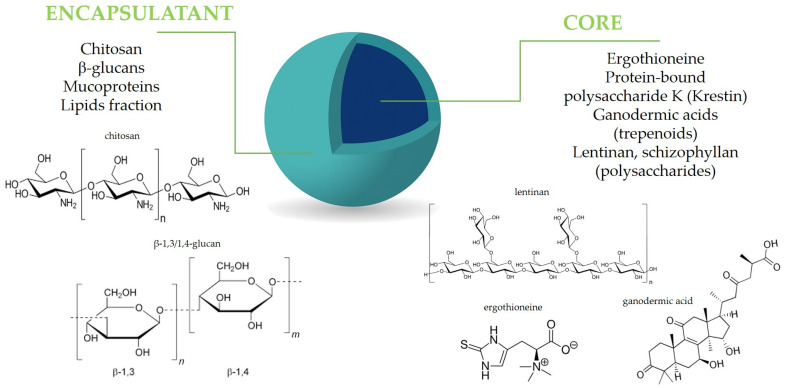
Mushroom components in microencapsulation design.

All active mushroom ingredients exhibiting antioxidant, immunomodulatory, and anti-inflammatory properties, including polysaccharides, polyphenols, terpenoids, and proteins, may be effectively close in a capsule structure (Figure 2). The encapsulation of fungal bioactive substances has, therefore, been extensively explored as a strategy to improve their functional and technological performance in food applications [91,92]. Additionally, mushroom-derived carriers, including polysaccharides, mucoproteins, and chitosan, present significant advantages for microencapsulation applications. Their ability to enhance stability, improve bioavailability, and provide controlled release makes them valuable materials for various industries [93,94,95,96]. Microencapsulation is still a developing method, continuously seeking new solutions and perspectives to enhance food production. Given the trend of using natural ingredients to replace synthetic additives, certain natural materials can serve as effective encapsulating agents. Materials with special pro-health properties, such as residues from the industrial production of mushrooms, are also of interest in microencapsulation. These residues include, for example, polysaccharides like β–glucans and ergosterol [97,98]. The combination of conventional wall materials with bioactive compounds that possess inherent antioxidant activity can enhance the stability of microcapsules and maintain bioactivity during drying and storage, and, finally, after being introduced to food, affect its pro-health properties [99]. Below, we mention some examples of successful technologies resulting in the mushroom-based microcapsules and their use for the improvement of the antioxidant and/or anti-inflammatory properties of food.

Ribeiro et al. [100] prepared microcapsules using spray-drying techniques with maltodextrin as the encapsulating material. The core consisted of lyophilized extracts of sticky bun mushroom (Sl; *Suillus luteus*) and shaggy cap (Ca; *Coprinopsis atramentaria*), as well as their combination in a 1:1 ratio (to prove the role of bioactive components interactions in the tailoring of antioxidant capacity). The study revealed that microcapsules from the studied mushrooms resulted in higher antioxidant activity, reflected in an IC50 value of 2.13 mg/mL for *Suillus luteus* and 2.36 mg/mL for *Coprinopsis atramentaria*, compared to their free extracts, which showed IC50 values of 2.86 mg/mL and 4.62 mg/mL, respectively, in the DPPH test. The co-encapsulation of the mixture of extracts (1:1) showed that components of both mushrooms acted synergistically. The microparticles (Sl:Ca (1:1)) as well as free-form extracts (Sl:Ca (1:1)) were then incorporated into cottage cheese. The results for the antiradical activity and reducing power demonstrated that the incorporation of free extracts initially resulted in higher antioxidant activity (an IC50 value of 84 mg/mL in the DPPH test), which significantly declined after seven days (162 mg/mL). In contrast, cottage cheese enriched with microencapsulated extracts, despite showing lower initial antioxidant activity (133 mg/mL), exhibited an increase after seven days (97 mg/mL). Similar trends were observed in the reducing power assay, reinforcing the protective effect of microencapsulation in preserving antioxidant properties over time. The authors suggest that this behavior results from the protection of antioxidants by the maltodextrin matrix and their gradual release into the product during storage. Button mushroom (*Agaricus bisporus*) alcoholic extracts were microencapsulated using spray-drying, with maltodextrin crosslinked with citric acid serving as the encapsulating material. Additionally, a thermal treatment was applied after atomization (in an oven at 140 °C for 4 h) to define the stability of additives. The obtained microcapsules were then incorporated into yoghurt. The incorporation of free extracts and thermally untreated microcapsules initially resulted in enhanced antioxidant activity (IC50 values: 214 and 272 mg/mL); however, this activity decreased significantly after seven days (IC50 values: 248 and 314 mg/mL). In contrast, thermally treated microencapsulated extracts exhibited a steady increase in antioxidant activity over the same period (IC50 values at day 0: 106 mg/mL; day 7: 48.7 mg/mL). Furthermore, the obtained *A. bisporus* extract and the produced microcapsules were evaluated for their radical scavenging (DPPH test) and anti-inflammatory activity (LPS-induced NO production in RAW 246.7 macrophages). As expected, raw extracts demonstrated the highest radical scavenging activity (IC50 = 4.3 mg/mL), followed by the thermally treated microencapsulated extract with an average IC50 of 13.0 mg/mL. Regarding anti-inflammatory activity, raw extracts displayed an IC50 of 250 µg/mL, while the other samples exhibited no significant activity [101]. Fogarasi et al. [102] compared Vienna sausages enriched with an acidic aqueous extract of porcini mushroom (*Boletus edulis*) (VSe) and microcapsules (VSm) prepared by spray drying of blends containing the extract and microcrystalline cellulose. Sausages with the polyphenol-rich extract and microcapsules had a higher polyphenol content compared to the control, with the highest polyphenol concentration found in VSe (569 µg/g). The addition of 1.5% microcapsules was not sufficient to significantly enrich the product with polyphenols; however, the peroxide value (PV) and TBARS levels determined during storage remained relatively low and below the recommended limits. Umaña et al. [99] microcapsulated (spray drying) sunflower oil by using maltodextrin as the primary encapsulating material and replacing it (13.5% *w*/*w* dry matter) with a mushroom concentrate from button mushroom (*Agaricus bisporus*) (a by-product rich in polysaccharides obtained after ergosterol extraction). The study demonstrated that the selected shell material was a valuable source of polyphenols, residual ergosterol (approximately 10%), and β-glucans (54% of total glucans). Additionally, the antioxidant activity of the mushroom concentrates effectively protected sunflower oil from oxidation during spray drying and slowed the oxidation process during storage (35 °C, 50% relative humidity). However, in the later stages of storage (after 150 days), the effectiveness of oxidation prevention decreased, as evidenced by a significant reduction in linoleic acid content (from 58% to 41%). Ergosterol, when combined with the mushroom concentrate, was better preserved and contributed to improved oxidative stability. Even though storage of these microcapsules resulted in a 95% decrease in ergosterol content after 150 days, in the capsules without the mushroom concentrate, it was degraded after only 2 days. A similar study conducted by Gunel [103] involved the preparation of spray-dried microcapsules of apricot kernel oil. As an encapsulating material, a mixture of maltodextrin and 5% (*w*/*w*) of a by-product derived from ergosterol extraction from button mushroom (*Agaricus bisporus*) was used as a substitute for emulsifiers. The study demonstrated that using mushroom by-product emulsions in spray drying resulted in a high product yield (63%). Due to the high antioxidant properties of the mushroom by-product, oxidation reactions were slowed down. Total oxidation values (TOTOX) for microcapsules containing the mushroom by-product were 37 after 10 days of storage at 60 °C, whereas non-microencapsulated apricot kernel oil oxidized after just 5 days, reaching a TOTOX value of 41. This study confirmed that the addition of mushroom-derived ingredients to microencapsulated powders can delay oxidative degradation.

Bušić et al. [104] prepared hydrogel microcapsules containing polyphenols isolated from Reishi (*Ganoderma lucidum*) using an ion exchange technique. They used three types of encapsulants: sodium alginate (A—control), alginate mixed with whey protein isolate (A-WPI), and alginate mixed with zein (A-Z) in an 80:20 (*w*/*w*) ratio. The extracted fraction contained 1.45 mg GAE/g polyphenols, as determined using the Folin–Ciocalteu reagent. The prepared hydrogels were analyzed for their retention of antioxidant capacity using ABTS and DPPH tests. The highest retention in the ABTS assay was observed for A-WPI (68%), followed by A-Z (63%) and A (61%). In contrast, in the DPPH assay, the best results were obtained for A-Z (58%), followed by A-WPI (54%) and A (47%). The use of selected encapsulating materials allowed for a slower release of polyphenols while preserving the antioxidant capacity of the formulated alginate-based hydrogels. The powder extract of β-glucan isolated from oyster mushroom (*Pleurotus ostreatus*) was microencapsulated using two different techniques: spray-drying (SD) and freeze-drying (FD), with maltodextrin as the encapsulant. Both techniques effectively preserved the β-glucan, with encapsulated samples retaining approximately 71% compared to 59% in the non-encapsulated β-glucan powder. A similar trend was observed for the total polyphenol and phenolic acid content. The spray-drying technique resulted in 3.4 mg GAE/g and 3.8 mg caffeic acid equivalents (CAE)/g, respectively, while the control sample contained 2.8 mg GAE/g and 3.6 mg CAE/g. The obtained powders exhibited the same antioxidant activity in the DPPH test (approximately 14%) compared to the control. However, spray-drying proved to be more effective in encapsulating the β-glucan powder [105].

Moreover, some studies showed that mushrooms’ polysaccharides (e.g., chitosan, beta-glucans, mannans, and galactomannans) and mucoproteins may be successfully used as encapsulating agents, improving process efficiency, controlling the release of ingredients, and maintaining their high stability and functionality. Chitosan, a deacetylated derivative of chitin obtained from mushroom cell walls, has been extensively used in microencapsulation due to its biocompatibility, film-forming ability, antimicrobial properties, and controlled release kinetics [106,107].

Gallotti et al. [108] used a mixture of maltodextrin and acacia gum (as a control) along with two extracts—W (from washed solids) and UW (from unwashed solids)—derived from *Pleurotus ostreatus*, containing β-glucans and proteins, as shell material to encapsulate sunflower oil. The total glucan content in the extracts was 22 g/100 g d.w. for the W extract and 27 g/100 g d.w. for the UW extract. Their antioxidant activity, measured using the ferric reducing antioxidant power (FRAP test), was 63.54 μmol Fe^2+^/100 g d.w. for the W extract and 46 μmol Fe^2+^/100 g d.w. for the UW extract. The resulting powders, obtained using the spray-drying technique, demonstrated their ability to protect vitamin E, as the α-tocopherol content in powders containing β-glucans was two times higher than in the control sample. Additionally, the encapsulated polyunsaturated fatty acids were better protected against oxidation, as indicated by a lower conjugated diene (CD) content (8 compared to 13 in the control sample). Vanden Braber et al. [109] used 0.5% glucosamine solutions (chitosan derivative modified using the Maillard reaction) (GACh) and 0.5% chitosan (Ch) as shell materials for encapsulation of quercetin. They showed that spray-dried “empty” powders already had antioxidant activity against reactive oxygen species (the higher activity was observed for microcapsules based on GACh, compared to those with the unmodified chitosan (Ch-MC)). The addition of quercetin enhanced these activities, although empty microcapsules themselves contributed significantly to radical scavenging properties (30–57% against HO^•^ and 70–80% against O^2•−^). In turn, both control samples and those with flavonoid showed approximately three-fold lower scavenging capacity against HO^•^ than against O^2•−^. Moreover, microcapsules formulated with the GACh derivative exhibited faster quercetin release under gastric conditions (almost two-fold) compared to those made with native Ch. The essential oil of clove buds was encapsulated in nanoparticles using a two-step emulsion–ionic gelation technique, with chitosan (1% solution) as the encapsulant. The microcapsules were prepared with a chitosan-to-oil extract ratio of 1:0.5. The study demonstrated that the antioxidant activity, analyzed using the DPPH test, was significantly higher in chitosan-loaded nanoparticles than in free, non-encapsulated extracts. In microcapsules containing chitosan, antioxidant activity ranged from 15.9% to 71.8%, compared to 15.4% to 60.4% in free extracts. Moreover, nanoparticles with added chitosan exhibited strong antibacterial activity, particularly against *L. monocytogenes* and *S. aureus*. As a result, the use of chitosan as a wall material for microencapsulation is recommended for applications in the food industry [107]. Also, Zhang et al. [110] used chitosan as an encapsulant to enclose quercetin in microcapsules using the ionic gelation method. The prepared chitosan nanoparticles were studied for their DPPH scavenging potential, showing a rapid increase in antioxidant activity, reaching values close to 80%. This ability was dependent on the concentration of microcapsules. A similar trend was observed in the reducing power test, where the reducing power of quercetin-loaded nanoparticles increased with concentration, as evidenced by higher absorbance in the analyzed samples. The formed microcapsules effectively preserved the antioxidant activity of quercetin, confirming the efficiency of its microencapsulation in chitosan. A similar study by Zhang et al. [111] involved the preparation of microcapsules using sulfated β-glucan-chitosan (DS-CS) isolated from *Hericium erinaceus* as wall material for resveratrol (RES) nanoencapsulation using the electrostatic self-assembly method. The study demonstrated that the formulated nanoparticles showed anti-inflammatory activity, as evaluated in LPS-induced RAW264.7 macrophages, by inhibiting the production of NO, IL-1β (interleukin-1 beta), IL-6 (interleukin-6), and TNF-α. The inhibition of NO release was dose-dependent. It is worth noting that both control samples, i.e., free RES (core material used for encapsulation) and “empty” chitosan nanoparticles (DS-CS NPs), as well as resveratrol-loaded β-glucan-chitosan nanoparticles (DS-CS-RES NPs), suppressed NO production at concentrations ranging from 2.5 to 50 μg/mL. However, the highest inhibitory effect was observed for the DS-CS-RES NPs. Moreover, DS-CS-RES NPs significantly inhibited the secretion of TNF-α, IL-6, and IL-1β at 50 μg/mL compared to the control. The increase in anti-inflammatory activity observed for the DS-CS-RES NPs suggests a potential synergistic effect between chitosan microcapsules and resveratrol. DS-CS-RES NPs had better anti-inflammatory activity than DS-CS NPs and RES alone. Additionally, the DS-CS-RES NPs exhibited improved bioavailability and increased cellular uptake, confirming their potential as an effective delivery system for resveratrol.

The microencapsulation of mushroom-derived bioactives, such as polysaccharides (e.g., β-glucans), polyphenols, terpenoids, and proteins, offers significant potential to enhance the functionality of food additives, improve their stability, and support health benefits. This technique is especially valuable for boosting bioavailability and ensuring controlled release of substances with antioxidant, immunomodulatory, and anti-inflammatory properties. Additionally, mushrooms themselves serve as excellent sources of encapsulating materials, including polysaccharides, mucoproteins, and chitosan, which contribute additional health-promoting properties. Various food products, such as dairy, oils, sausages, and bakery, have benefited from the incorporation of mushroom-derived microencapsulated additives. A key advantage of mushroom-based microcapsules is their ability to replace synthetic food additives with naturally sourced, functional ingredients. However, despite these benefits, microencapsulation remains an evolving technique, and its long-term effectiveness remains a challenge. Ongoing research into optimizing encapsulation methods and exploring new mushroom-based materials is expected to lead to more efficient and sustainable food production processes in the future.

## 4. Conclusions and Future Perspectives

In recent years, mushrooms have become versatile and sustainable ingredients in the food industry. Mushrooms have become recognized as enhancing organoleptic properties and nutritional value and as having significant functional, health-promoting benefits. This review summarizes antioxidant and anti-inflammatory properties; however, it should be remembered that ingredients exhibiting these activities, such as polysaccharides or terpenoids, are also recognized as having a wider range of pro-health benefits. Additionally, the growing demand is fueling eco-friendly innovation and sustainability efforts, which is reflected in biotechnological approaches aimed at obtaining mycoproteins, polysaccharides, and other functional components of mushroom origin. Beyond these aspects of mushroom application, the industry has seen a surge in innovative mushroom-based products that differ in form and expected functionality. To meet the high demands of the modern food industry, mushroom-based additives—along with their enhanced nutritional and health benefits—must also consider factors such as sensory acceptance, shelf life, technological and processing changes, as well as legal, regulatory, and safety aspects.

Globally, the legal framework surrounding mushroom-derived additives is complex and diverse. Regulatory approaches differ depending on whether the additive is considered a novel food, a dietary supplement, or a pharmaceutical ingredient. These differences significantly impact the development and commercialization of mushroom-based technologies. In addition to traditionally consumed mushrooms (legally marketable), additives are often based on species or components that are not typically consumed but are known, e.g., from folk medicine. This aspect is related to legal regulations, which, without prior assessment of broader population effects, may limit or even exclude the application of these products in human nutrition. For example, in the European Union, the new Regulation (EU) 2015/2283 classifies this product category as a Novel Food, defined as food that had not been consumed significantly in the EU before 15 May 1997. It can be newly developed, innovative food, food produced using new technologies and production processes, or food that has been traditionally eaten outside of the EU. According to these regulations, novel foods must be safe for consumers, properly labelled (so as not to mislead consumers), and must not differ from traditional food (if it replaces others) in a way that would make its consumption nutritionally disadvantageous. In light of this, pre-market authorization requires scientists and industry to thoroughly evaluate new mushroom-based products in terms of their potential impact on consumer well-being and health. On the other hand, in other countries, e.g., the United States, new food ingredients must be evaluated for their safety before they are allowed to be marketed into the food supply (a long-standing approach aimed to confirm the Generally Recognized as Safe (GRAS) status). Moreover, in China, the pre-market approval of new food raw materials (counterpart of “novel food” from the EU) is conducted by the National Health and Family Planning Commission [112].

Despite promising results regarding the desired activities of mushrooms and mushroom-based additives, including their antioxidant and anti-inflammatory properties, many issues still require regulation as well as comprehensive and in-depth research. We showed that mushroom-based additives may be produced using different technological approaches, covering effective and efficient extraction techniques, and designing new matrices and forms. An important aspect is the validation of materials by clearly defining the species used, cultivation conditions, post-harvest processing impact, and standardized extraction methods. All these factors significantly influence the bioactive component content, ultimately affecting the health benefits of food additives derived from these materials. There is also still a lack of information regarding the bioaccessibility/bioavailability of mushroom antioxidants, which may undergo degradation and transformation during digestion and be further metabolized. However, it is proven that bioaccessibility is usually significantly improved if food additives containing isolated fractions are applied to food compared to, e.g., dried fruit bodies. Much evidence also shows that the functionality of mushroom-derived compounds is created by possible interactions with the food matrix. The production technology often played a crucial role in maintaining a high retention of antioxidants and anti-inflammatory compounds, and the powders exhibiting similar activities showed different effects after being introduced into the different products. Finally, the stability and shelf life of additives also play an important role, and the mushroom antioxidant and anti-inflammatory activity are usually dramatically altered by the time of purchase. All these criteria have to be met during the design and confirmation of the use of food additives containing antioxidants of mushroom origin.

In conclusion, mushroom-derived food additives with antioxidant and/or anti-inflammatory properties hold great promise as natural and effective functional ingredients for 21st-century food. Their purpose extends beyond providing nutrients; however, they are obligated to enhance both the health and well-being of modern communities. Although significant progress has been made, further research is still needed to develop and deliberately design their production technologies, allowing desirable functionality as well as the opportunity to evaluate their effect on the quality of food products and the resulting health benefits.

## Supplementary Materials

The following supporting information can be downloaded at: https://www.mdpi.com/article/10.3390/antiox14050519/s1.
