# Supplementary material for: Antioxidant and Anti-Inflammatory Properties of Mushroom-Based Food Additives and Food Fortified with Them—Current Status and Future Perspectives
Source: Antioxidants (Basel). 2025 Apr 26;14(5):519. doi: 10.3390/antiox14050519 (PMC12108364; doi:10.3390/antiox14050519)
Supplement: Supplementary file 1 [file antioxidants-14-00519-s001.zip › antioxidants-3589824-supplementary.pdf]

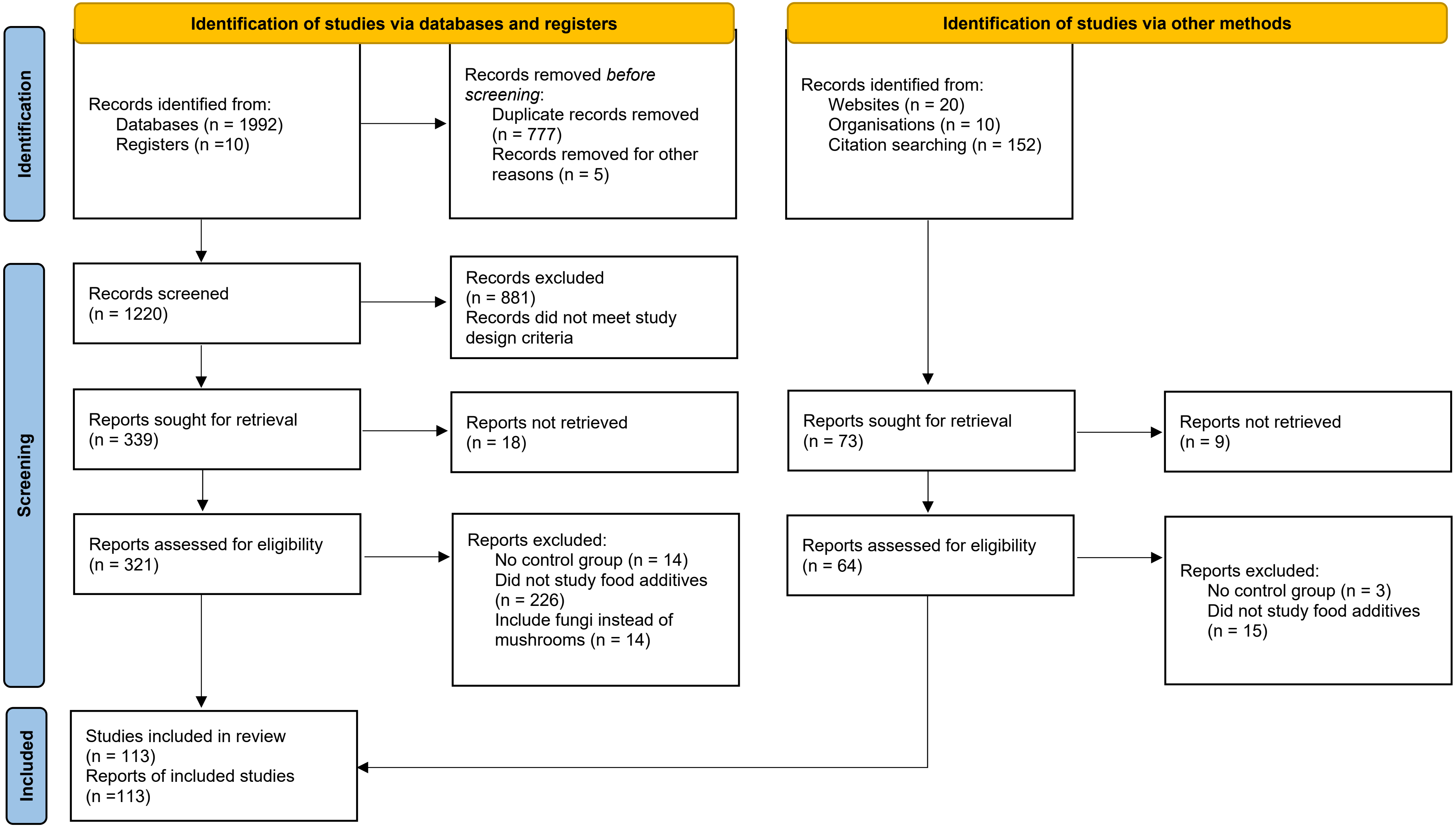

Figure S1. PRISMA flow diagram for “Antioxidant and Anti-Inflammatory Properties of Mush-room-Based Food Additives and Food Fortified with Them – Current Status and Future Perspectives”
